# *Lactobacillus reuteri* TSR332 and *Lactobacillus fermentum* TSF331 stabilize serum uric acid levels and prevent hyperuricemia in rats

**DOI:** 10.7717/peerj.11209

**Published:** 2021-05-03

**Authors:** Yi-Wei Kuo, Shih-Hung Hsieh, Jui-Fen Chen, Cheng-Ruei Liu, Ching-Wei Chen, Yu-Fen Huang, Hsieh-Hsun Ho

**Affiliations:** Research and Development Department, Glac Biotech Co., Ltd., Tainan, Taiwan

**Keywords:** Probiotics, *Lactobacillus*, Purine metabolism, Hyperuricemia, Gout

## Abstract

**Background:**

Uric acid (UA) is the end product of purine metabolism in the liver and is excreted by the kidneys. When purine metabolism is impaired, the serum UA level will be elevated (hyperuricemia) and eventually lead to gout. During evolution, humans and some primates have lost the gene encoding uricase, which is vital in UA metabolism. With the advances of human society, the prevalence of hyperuricemia has dramatically increased because of the refined food culture. Hyperuricemia can be controlled by drugs, such as allopurinol and probenecid. However, these drugs have no preventive effect and are associated with unpleasant side effects. An increasing number of probiotic strains, which are able to regulate host metabolism and prevent chronic diseases without harmful side effects, have been characterized. The identification of probiotic strains, which are able to exert beneficial effects on UA metabolism, will provide an alternative healthcare strategy for patients with hyperuricemia, especially for those who are allergic to anti-hyperuricemia drugs.

**Methods:**

To elicit hyperuricemia, rats in the symptom control group (HP) were injected with potassium oxonate and fed a high-purine diet. Rats in the probiotic groups received the high-purine diet, oxonate injection, and supplements of probiotic strains TSR332, TSF331, or La322. Rats in the blank control group (C) received a standard diet (AIN-93G) and oxonate injection.

**Results:**

Purine-utilizing strains of probiotics were screened using high-pressure liquid chromatography (HPLC) in vitro, and the lowering effect on serum UA levels was analyzed in hyperuricemia rats in vivo. We found that *Lactobacillus reuteri* strain TSR332 and* Lactobacillus fermentum* strain TSF331 displayed significantly strong assimilation of inosine (90%; *p* = 0.00003 and 59%; *p* = 0.00545, respectively) and guanosine (78%; *p* = 0.00012 and 51%; *p* = 0.00062, respectively) within 30 min in vitro. Further animal studies revealed that serum UA levels were significantly reduced by 60% (*p* = 0.00169) and 30% (*p* = 0.00912), respectively, in hyperuricemic rats treated with TSR332 and TSF331 for 8 days. Remarkably, TSR332 ameliorated the occurrence of hyperuricemia, and no evident side effects were observed. Overall, our study indicates that TSR332 and TSF331 are potential functional probiotic strains for controlling the development of hyperuricemia.

## Introduction

In addition to their crucial roles in DNA and RNA, purines are also significant components of several other important biomolecules, including ATP, GTP, cyclic AMP, NADH, and coenzyme A (Rosemeyer, 2004). Purine neurotransmitters, which act upon purinergic receptors, are purine derivatives that maintain physical functions in species that have a nervous system (Burnstock, 2017). High-purine concentrations are found in meat and meat products, especially in internal organs such as the liver and kidneys. High-purine foods such as sardines, brains, meat extracts, herring, mackerel, scallops, beer, and gravy usually have rich flavors. Moderate-purine foods are red meat and some flavorful vegetables such as asparagus, cauliflower, spinach, mushrooms, green peas, beans, and grains (Beyl Jr, Hughes & Morgan, 2016). Purine metabolism takes place in the liver, and the end product is uric acid (UA). When UA is insufficiently eliminated via urine, blood UA levels are persistently elevated, which can result in gout. Most animals produce uricase, which degrades UA, and thus rarely experience gout (Wu et al., 1994). However, humans and apes have mutations in the liver uricase genes and therefore produce inactive UA oxidase (Agudelo & Wise, 2001). Therefore, hyperuricemia is more common in humans than in other mammals.

A total of 360 µmol/L (6 mg/dL) and 400 µmol/L (6.8 mg/dL) of UA in blood is the upper limit of the normal range for women and men, respectively (Chizynski & Rozycka, 2005). Hyperuricemia has various potential origins: diet, impaired kidney function, fasting, drugs, or tumor (Cirillo et al., 2006; Howard, Jones & Pui, 2011). In Taiwan, more than 20% of men above the age of 19 years and approximately 17% of women above the age of 45 years have hyperuricemia. One in 10 patients with hyperuricemia develops gout, which is a form of inflammatory arthritis (Dalbeth, Merriman & Stamp, 2016). Studies have mapped the biological relationship of UA with other diseases, such as vascular damage, hypertension, endothelial dysfunction, and renal disease (Soltani et al., 2013; Zoccali & Mallamaci, 2013). Causes of hyperuricemia can be classified into three types: decreased excretion of UA, increased production of UA, and mixed type (Yamamoto, 2008). Although hyperuricemia medicine is available, mild side effects, such as itchiness and rash, are commonly seen, and rare lethal side effects have been reported in certain populations (Dewan & Quinonez, 2010; Fernando & Broadfoot, 2010). Moreover, medicine needs to be used with caution because of active drug interactions with other medicines (Brown, 1993; Utsunomiya et al., 2010). Both lifestyle changes and medications may decrease UA levels; however, a strict restriction on purine-containing food is nearly impossible. Therefore, an increasing number of studies have focused on seeking natural supplementary for hyperuricemia.

Probiotic products have drawn more and more attention by their beneficial effects on metabolic disorders. The microbiota is unique in every individual and can be dynamically changed by daily life. Any change in the mental or physical condition of the host can strongly affect the gut bacterial composition (Thursby & Juge, 2017). Unhealthy eating habits can tilt the microbiome balance, and an imbalance in microbiota composition may predispose to disease. Increasing evidence shows that the gut microbiota is involved in the host’s energy metabolism (Cani & Delzenne, 2009). Gut microbiota consists of bacteria, archaea, and fungi; however, not every microorganism plays a role in the host metabolism. By screening among the population, specific strains of probiotics, which can actually effect the metabolism in the host, can feasibly be obtained. For instance, an evidence has shown positive effect of probiotics in hypercholesterolemic animal models (Taranto et al., 1998), and the bacteria was hypothesized to interfere cholesterol absorption from the gut by deconjugating bile salts, or by directly assimilating cholesterol. However, the beneficial influence of probiotics on the host’s metabolism may be strain-specific, and the result can be different in animal and human studies, or in different groups of human studies (St-Onge et al., 2002; Pereira & Gibson, 2002).

By screening and characterization of purine nucleoside degrading lactic acid bacteria it should be possible to obtain probiotic strains that are capable of alleviating UA production from dietary purine (Li et al., 2014; Garcia-Arroyo et al., 2018; Wang et al., 2019; Yamanaka et al., 2019). Although several probiotic strains have been patented (EP2476747A1; EP2457576A1; EP1649863A1; CN101932697B) for their beneficial effects on hyperuricemia, the efficiencies of these strains are neither comparable nor universal. More UA-lowering probiotic strains are needed for better understanding on the role of microbiome in UA metabolism. In this study, analysis of purine metabolism was carried out in 24 probiotic strains to screen for UA-lowering effects. Three of the probiotic strains were tested in a hyperuricemia animal model. The therapeutic and preventive effects of the strain TSR332 were further investigated in moderate and acute hyperuricemia animal models. The symptom was induced for 8 days in the moderate animal model, and for 14 days in the acute animal model.

## Materials & Methods

### Probiotic cultivation and analysis of purine nucleoside assimilation in vitro

Nineteen probiotic strains, including TSR332 (*Lactobacillus reuteri*); TSF331, L-18, and L-319 (*Lactobacillus fermentum*); La-322 (*Lactobacillus amylovorus*); TSP05, L-29, and L-335 (*Lactobacillus plantarum*); L-120 (*Lactobacillus paracasei*); L-87 (*Lactococcus lactis* subsp*. lactis*); L-39 (*Bifidobacterium animalis* subsp*. lactis*); L-85 and L-134 (*Lactobacillus rhamnosus*); L-243 (*Streptococcus thermophilus*); L-69 (*Bifidobacterium animalis subsp. lactis*); L-291 (*Lactobacillus acidophilus*); L-9 (*Lactobacillus casei* subsp*. casei*); Bv-889 (*Bifidobacterium breve*); and L-2 (*Lactobacillus gasseri*), were provided by Glac Biotech Co., Ltd. (Tainan, Taiwan). TSR332 and TSF331 were isolated from human gut, and TSP05 was isolated from kimchi. Four probiotic strains, including BCRC14045 and BCRC14060 (*Bifidobacterium breve*); and BCRC14619 and BCRC17616 (*Lactobacillus gasseri*), were purchased from the Bioresource Collection and Research Center, Hsinchu City, Taiwan. One probiotic strain, LGG (*Lactobacillus rhamnosus*), was obtained from Chr. Hansen Inc. (Milwaukee, WI). The probiotics were cultured in de Man, Rogosa & Sharpe broth containing 0.05% cysteine for 20 h.

To measure purine assimilation, 10^9^ probiotic cells were isolated and incubated in one mL of trisodium phosphate solution (0.1 M) containing 1.25 mM inosine or guanosine for 30 min. Then, 0.9 ml of supernatant was collected and mixed with 0.1 mL of perchloric acid to stop purine degradation. Inosine, guanosine, hypoxanthine, and guanine levels in the supernatant were analyzed using high-pressure liquid chromatography (HPLC). The degradation rate of nucleosides was calculated as (standard _total area of nucleosides_ – sample _total area of nucleosides_) / standard _total area of nucleosides_ (%). Purine base generation was calculated as sample _total area of base_ / standard _total area of nucleosides_.

### HPLC

Purine and purine bases were analyzed using an Altus HPLC instrument equipped with a photodiode array detector (PerkinElmer, MA, USA). The analysis column was a Kinetex 2.6 µm F5 100A (150 × 3 mm, Phenomenex, CA, USA). The mobile phase solutions were methanol and H_2_O. The samples (3 µL) were injected into the HPLC column at 28 °C, with a flow rate of 0.25 mL/min. The optical density at 254 nm was measured in the eluted samples. The results were analyzed using the Empower 3 software (Waters, MA, USA).

### Animal treatment

The experiments were carried out according to regulations controlled by the Animal Research Committee of HKU; all experimental protocols were approved by the Ethical Committee of the Hungkuang University (approved affidavit No.: HK-P-10512). Animal studies were carried out in the Department of Food Science and Technology, Hung Kuang University. Thirty-day old male Wistar rats were purchased from BioLASCO Taiwan Co., Ltd and were housed one rat per cage at the Laboratory Animal Center, Hungkuang University (HKU). The animals arrived with initial weights of approximately 100 g and were given free access to sterilized water and food. The rats were housed under a 12-h light/dark cycle, at 22 ± 2 °C, and 62% ± 5% humidity. The rats were familiarized with human handling and the laboratory one week prior to the start of the experiments. All animals were healthy, and no signs of pain or distress were observed. No euthanasia was needed during the experiments. All animals were anesthetized using Zoletil/Xylazine before cervical dislocation. Animal experiments and protocols were in compliance with the Laboratory Animal Care and Use Guidelines published by the Taiwanese government.

### Hyperuricemia induction and experimental setup

To elicit hyperuricemia, the rats were fed a high-purine diet containing 87 g yeast extract (Sigma, St. Louis, MO) and 1.5 g ribonucleic acid from torula yeast (R6625; Sigma) to induce hyperuricemia. Subsequently,rats were injected intraperitoneally with potassium oxonate (0.35 mg/100 g body weight/day) dissolved in carboxymethylcellulose sodium solution (3 g/L). Bodyweight, UAs, and creatinine levels in the hyperuricemia model rats were analyzed at selected time points.

For the control group (C), rats received normal diets (AIN-93G) and oxonate injections. For the symptom control group (HP), rats received high-purine diets and oxonate injections. For probiotic groups, rats receive high-purine diets, probiotics supplements, and oxonate injections. The probiotic strains were given via oral 1,000-µL gavages containing 10^9^ CFU/kg/day of each probiotic strain in 0.85% NaCl. Eight rats were examined per group.

**Figure 1:**
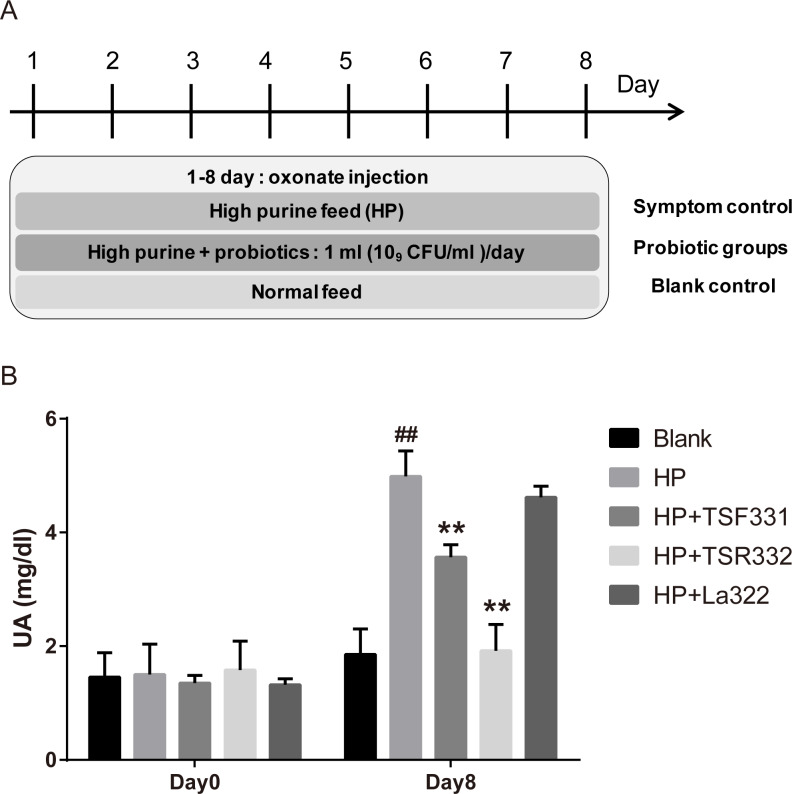
Preliminary tests of TSF331, TSR332, and La322 in hyperuricemic rats. Five groups of Wistar rats were set up, and the rats were daily injected intraperitoneally with potassium oxonate. The blank control group was fed a regular diet, and the four other groups were fed a high-purine diet to induce hyperuricemia. Three of the hyperuricemic groups were treated with 10^9^ CFU/ml of TSF331, TSR332 and La322, respectively, in 0.85% NaCl daily for 8 days. Blood serum was collected and tested for UA before and after treatments on days 0 and 8. Data are the mean ±  SD. ^##^*p* < 0.01 blank vs. hyperuricemic group, ^∗∗^*p* < 0.01, hyperuricemic vs. probiotics-treated group, one-way ANOVA.

To investigate the effect of TSF331, TSR332, and La322 in hyperuricemia rats, experiments were carried out for 8 days, as shown in Fig. 1A. Blood samples were collected on days 0 and 8.

**Figure 2:**
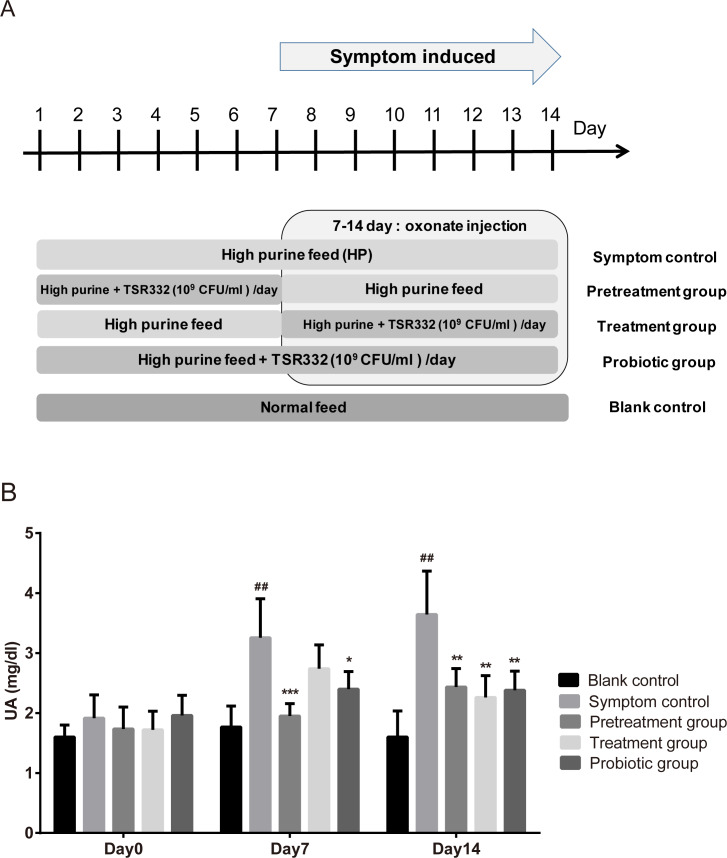
Pretrestment and treatment effects of TSR332 in hyperuricemic rats. Five groups of Wistar rats were set up and one blank control group was fed a regular diet. Four other groups were fed a high-purine diet, and the rats were daily injected intraperitoneally with potassium oxonate from day 7 to 14 to induce hyperuricemia. Three of the hyperuricemic groups were treated with 10^9^ CFU/ml of TSR332 in 0.85% NaCl for the designated periods (days 1–7, days 7–14, or days 1–14). Blood serum was collected and tested for UA levels on days 0, 7, and 14. Data are the mean ± SD. ^##^ *p* < 0.01 blank vs. hyperuricemic group, and ^∗^*p* < 0.05, ^∗∗^*p* < 0.01, ^∗∗∗^*p* < 0.005 hyperuricemic vs. TSR332-treated group, one-way ANOVA.

To investigate the preventive and therapeutic effects of TSR332, experiments were carried out, as shown in Fig. 2A. During days 1 through 6, TSR332 was administered daily in two groups: the prevention and combination groups. On day 7, all groups were injected with potassium oxonate for another 8 days. During days 7 through 14, TSR332 supplementation was stopped in the prevention group and was continued in the combination group. The therapeutic group was treated with TSR332, simultaneously with potassium oxonate injection, from day 7 to 14. Rats in the symptom control (HP) were given a high-purine diet from day 1 to 14 and a potassium oxonate injection from day 7 to 14. Blood samples were collected on days 0, 7, and 14.

**Figure 3:**
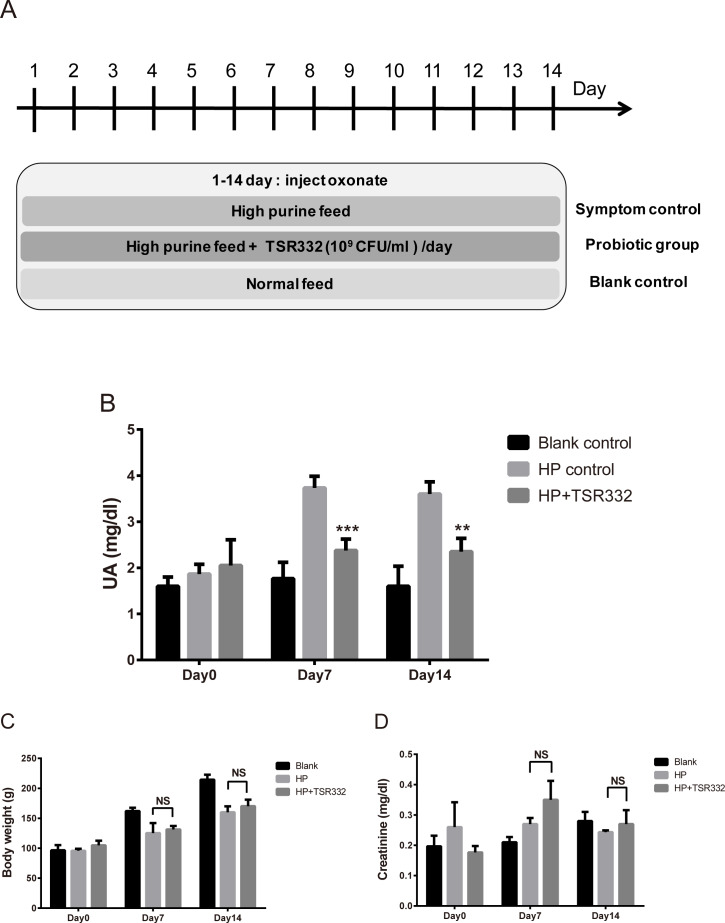
UA, body weight, and kidney function in acute hyperuricemic rats. Three groups of Wistar rats were set up, and the rats were daily injected intraperitoneally with potassium oxonate to induce uric acid production. The blank control group was fed a regular diet, and two other groups were fed a high-purine diet to induce hyperuricemia. One of the hyperuricemic groups was treated with 10^9^ CFU/ml of TSR332 in 0.85% NaCl daily for 14 days. Body weight was measured (B), and blood serum was collected to test for (A) UA and creatinine levels on days 0, 7, and 14. Data are the mean ± SD. ^#^*p* < 0.05, ^##^*p* < 0.01) blank vs. hyperuricemic group, ^∗∗^*p* < 0.01, ^∗∗∗^*p* < 0.005 hyperuricemic vs. TSR332-treated group, one-way ANOVA.

To investigate the therapeutic effect of TSR332 in rats with severe hyperuricemia, experiments were carried out for 14 days, as shown in Fig. 3A. Body weights were measured, and blood samples were collected on days 0, 7, and 14.

### Evaluation of body weight, creatinine, and UA

The animal body weight was measured, and blood samples were collected for UA and creatinine analyses on days 0, 7, and 14. One milliliter of blood was collected from the tail vein of each animal and the serum was obtained by centrifugation at 1350 × *g* for 5 min. UA levels were determined using UA stript#25 and an EasyTouch GCU multi-function monitoring system (Bioptik Technology, Inc., Miaoli County, TW). Creatinine levels were determined using LabAssay#290-65901 (Wako Chemicals Inc., VA, USA) and an Automated Clinical Chemistry Analyzer (FUJI DRI-CHEM 4000i; Fuji, Japan).

### Statistical analysis

Statistical analysis was performed using GraphPad Prism software. Data are presented as the mean ± standard deviation (SD) obtained from eight animals. Differences were evaluated using one-way ANOVA followed by Tukey’s range test for multiple comparisons. A *p* < 0.05 was considered statistically significant.

## Results

### *L. reuteri* TSR332 and *L. fermentum* TSF331 rapidly metabolize purine nucleosides, without the production of UAs

**Figure 4:**
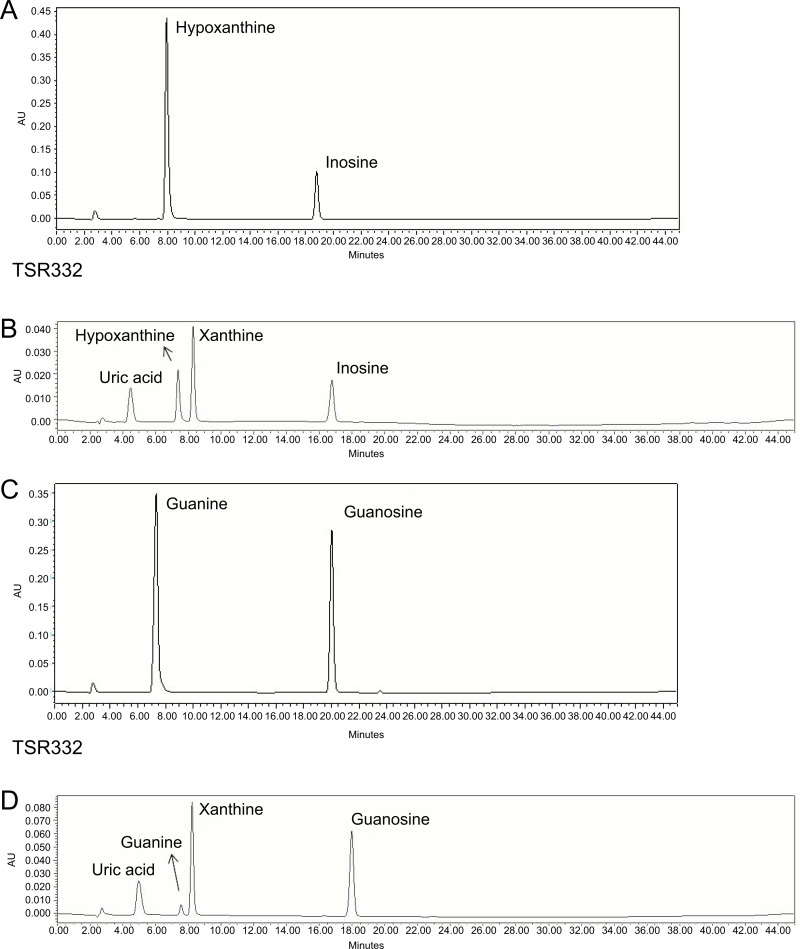
Resolution of purines and purine metabolites by HPLC analysis. (A) Inosine degradation and hypoxanthine production within 30 min by the probiotic strain, TSR332. (B) A mixture of inosine, xanthine, hypoxanthine, and uric acid was used as the standard solution for the inosine consumption test. (C) Guanosine degradation and guanine production within 30 min by TSR332. (D) A mixture of guanosine, xanthine, guanine, and uric acid was used as the standard solution for the guanosine consumption test. Standard curves were established using 0.625 mM, 1.25 mM, and 2.5 mM solutions.

To screen potential probiotic strains, 24 strains (Table 1) were tested for inosine and guanosine assimilation abilities based on HPLC detection. Compounds detected in inosine assimilation were inosine, xanthine, hypoxanthine, and UA. Compounds detected in guanosine assimilation were guanosine, xanthine, guanine, and UA. Purine degradation for inosine and guanosine by strain TSR332 (Figs. 4A, 4C) showed an apparent decrease in purines and an increase in purine derivatives when compared to that of standard solutions (Figs. 4B, 4D). HPLC results for all strains are summarized in Table 2. The majority of the 24 tested strains exhibited purine assimilation, with an average assimilation rate of 25.72% for inosine and of 20.54% for guanosine. Among them, strains TSR332 and TSF331 were found to metabolize purine nucleosides at rates higher than 50% within 30 min. In particular, TSR332 showed the highest rates of 90.05% and 78.02% for inosine and guanosine, respectively.

**Table 1:** List of probiotic strains used for purine assimilation analysis.

**Probiotic strain**	**Species name**
TSR332	*Lactobacillus reuteri*
TSF331	*Lactobacillus fermentum*
TSP05	*Lactobacillus plantarum*
L-319	*Lactobacillus fermentum*
La-322	*Lactobacillus amylovorus*
L-335	*Lactobacillus plantarum*
L-18	*Lactobacillus fermentum*
Bv-889	*Bifidobacterium breve*
L-2	*Lactobacillus gasseri*
L-69	*Bifidobacterium animalis* subsp*. lactis*
L-243	*Streptococcus thermophilus*
L-291	*Lactobacillus acidophilus*
L-120	*Lactobacillus paracasei*
L-39	*Bifidobacterium animalis* subsp*. lactis*
L-87	*Lactococcus lactis subsp. lactis*
L-134	*Lactobacillus rhamnosus*
L-9	*Lactobacillus casei* subsp*. casei*
L-29	*Lactobacillus plantarum*
L-85	*Lactobacillus rhamnosus*
LGG	*Lactobacillus rhamnosus*
BCRC14045	*Bifidobacterium breve*
BCRC14060	*Bifidobacterium breve*
BCRC14619	*Lactobacillus gasseri*
BCRC17616	*Lactobacillus gasseri*

**Table 2:** HPLC analysis of purine assimilation and metabolite generation in twenty-four probiotic strains.^a^

	Composition of the purine compounds				
Probiotic strains	Inosine (mM)	Hypoxanthine (mM)	Guanosine (mM)	Guanine (mM)	Xanthine (mM)/ uric acid (mM)
TSR332	1.126 ± 0.05	0.754 ± 0.049	0.975 ± 0.063	0.830 ± 0.272	0 ± 0.00/0 ± 0.00
TSF331	0.743 ± 0.082	0.497 ± 0.017	0.641 ± 0.038	0.327 ± 0.124	0 ± 0.00/0 ± 0.00
TSP05	0.603 ± 0.063	0.189 ± 0.017	0.569 ± 0.055	0.227 ± 0.105	0 ± 0.00/0 ± 0.00
L-319	0.528 ± 0.094	0.235 ± 0.009	0.316 ± 0.072	0.150 ± 0.097	0 ± 0.00/0 ± 0.00
L-322	0.465 ± 0.057	0.102 ± 0.004	0.223 ± 0.032	0.123 ± 0.098	0 ± 0.00/0 ± 0.00
L-335	0.381 ± 0.059	0.006 ± 0.000	0.200 ± 0.051	0.119 ± 0.095	0 ± 0.00/0 ± 0.00
L-18	0.357 ± 0.044	0.086 ± 0.009	0.283 ± 0.059	0.131 ± 0.096	0 ± 0.00/0 ± 0.00
Bv-889	0.320 ± 0.030	0.000 ± 0.000	0.987 ± 0.050	0.000 ± 0.000	0 ± 0.00/0 ± 0.00
L-2	0.303 ± 0.018	0.000 ± 0.000	0.221 ± 0.050	0.000 ± 0.000	0 ± 0.00/0 ± 0.00
L-69	0.283 ± 0.059	0.091 ± 0.009	0.113 ± 0.061	0.170 ± 0.098	0 ± 0.00/0 ± 0.00
L-243	0.219 ± 0.057	0.000 ± 0.000	0.052 ± 0.017	0.000 ± 0.000	0 ± 0.00/0 ± 0.00
L-291	0.177 ± 0.057	0.004 ± 0.001	0.053 ± 0.047	0.000 ± 0.000	0 ± 0.00/0 ± 0.00
L-120	0.142 ± 0.007	0.000 ± 0.000	0.027 ± 0.013	0.000 ± 0.000	0 ± 0.00/0 ± 0.00
L-39	0.127 ± 0.075	0.045 ± 0.009	0.135 ± 0.042	0.122 ± 0.095	0 ± 0.00/0 ± 0.00
L-87	0.114 ± 0.030	0.000 ± 0.000	0.060 ± 0.003	0.000 ± 0.000	0 ± 0.00/0 ± 0.00
L-134	0.101 ± 0.067	0.000 ± 0.000	0.060 ± 0.020	0.000 ± 0.000	0 ± 0.00/0 ± 0.00
L-9	0.082 ± 0.021	0.000 ± 0.000	0.078 ± 0.045	0.000 ± 0.000	0 ± 0.00/0 ± 0.00
L-29	0.051 ± 0.014	0.041 ± 0.003	0.102 ± 0.072	0.115 ± 0.095	0 ± 0.00/0 ± 0.00
L-85	0.050 ± 0.012	0.000 ± 0.000	0.072 ± 0.079	0.000 ± 0.000	0 ± 0.00/0 ± 0.00
LGG	0.079 ± 0.014	0.000 ± 0.000	0.068 ± 0.022	0.000 ± 0.000	0 ± 0.00/0 ± 0.00
BCRC14045	0.327 ± 0.045	0.095 ± 0.003	0.191 ± 0.048	0.121 ± 0.096	0 ± 0.00/0 ± 0.00
BCRC14060	0.307 ± 0.022	0.031 ± 0.002	0.305 ± 0.046	0.118 ± 0.095	0 ± 0.00/0 ± 0.00
BCRC14619	0.475 ± 0.023	0.019 ± 0.002	0.297 ± 0.074	0.111 ± 0.095	0 ± 0.00/0 ± 0.00
BCRC17616	0.354 ± 0.047	0.112 ± 0.004	0.133 ± 0.035	0.079 ± 0.109	0 ± 0.00/0 ± 0.00
control	0.000 ± 0.081	0.000 ± 0.000	0.00 ± 0.053	0.000 ± 0.000	0 ± 0.00/0 ± 0.00

**Notes.**

a Cells of the probiotic strains (Glac Biotech Co., Ltd., Tainan, Taiwan) were incubated with 1.25 mM inosine-guanosine solution at 37 °C for 30 min under shaking (140 rpm). The reaction was terminated by adding 0.1 M HClO_4_ into the supernatant to prevent further degradation. Fifty microliters of the mixture was injected into the HPLC column after 0.22-μ m filtration. The contents of hypoxanthine, guanine, and uric acid were calculated.

To identify downstream degradation products produced by the probiotic strains, the same solutions used in the above tests were further analyzed for purine metabolites by HPLC. The results are summarized in Table 2. Hypoxanthine and guanine were the two main components of the metabolites, and UA was not detected. TSR332 and TSF331 displayed the highest purine-metabolizing rates within 30 min. In a 1.25 mM inosine solution, 1.126 mM and 0.743 mM of inosine was degraded, and 0.754 mM and 0.497 mM of hypoxanthine was produced by TSR332 and TSF331, respectively. In a 1.25 mM guanosine solution, 0.975 mM and 0.641 mM of guanosine was degraded, and 0.083 mM and 0.327 mM of guanine was produced by TSR332 and TSF331, respectively (Table 2).

**Figure 5:**
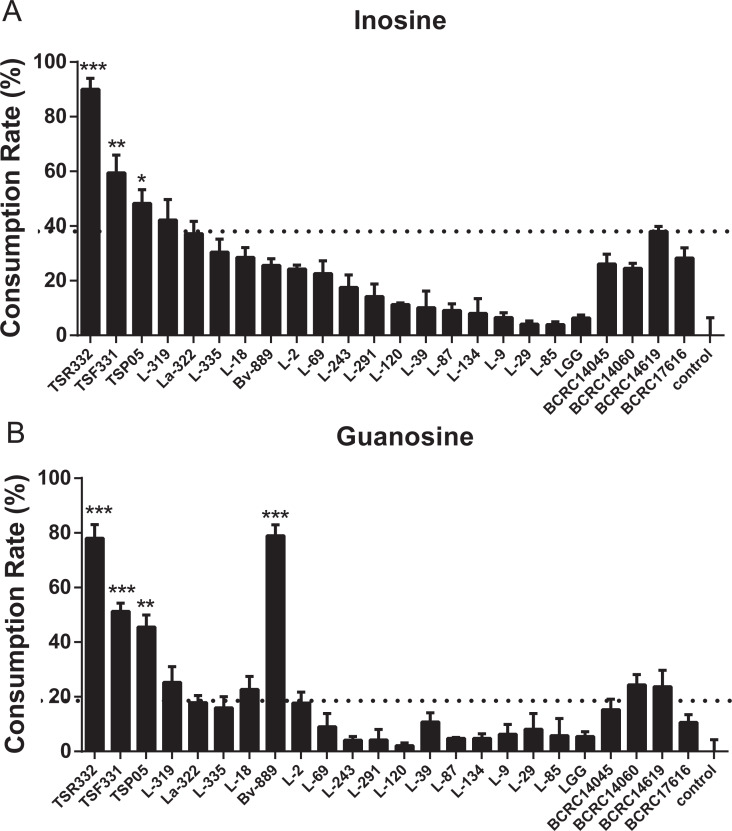
In vitro purine assimilation assays of inosine and guanosine in probiotic strains. (A) Inosine assimilation rates in the probiotic strains within 30 min. (B) Guanosine assimilation rates in the probiotic strains within 30 min. Data are the mean ± SD. ^∗^*p* < 0.05, ^∗∗^*p* < 0.01, ^∗∗∗^*p* < 0.001 vs. strain La322, one-way ANOVA. The dotted line represents the assimilation rate in La322.

Inosine and guanosine assimilation rates were compared side by side for the 24 strains. The purine assimilation ability varied widely, from 2.15% to 90.05%, among the strains (Fig. 5, Table S4). Inosine and guanosine assimilation rates for L-85 were very low (3.98% and 5.77%, respectively). Moderate inosine and guanosine assimilation rates were observed for La-322 (37.17% and 17.85%, respectively). Like TSR332, TSF331 exhibited high inosine and guanosine assimilation rates, of 59.43% and 51.26%, respectively (Fig. 5). The two outstanding stains, TSR332 and TSF331, together with one moderately performing strain, La322, were selected for the animal study.

### *L. reuteri* TSR332 and *L. fermentum* TSF331 stabilize the level of UA in hyperuricemia model rats

To preliminarily examine the effects of TSR332, TSF331, and La322 on serum UA levels in rats, hyperuricemia was induced by intraperitoneal injection of potassium oxonate and a high-purine diet. Probiotic strains were supplemented to hyperuricemia rats for 8 days (Fig. 1). The outline of this study is presented in Fig. 1A. The blank control group was fed a regular diet, and 4 other groups were fed a high-purine diet to increase the purine uptake from food. One hyperuricemia group served as the symptom control (HP), and three of the hyperuricemia groups were supplemented with probiotic strains daily for 8 days. Blood samples were taken on days 0 and 8. The results showed that hyperuricemia was induced successfully as soon as potassium oxonate was injected, and serum UA levels were significantly (^##^*p* = 0.00598) higher in the HP group (4.99 mg/dL) than in the control group (1.86 mg/dL) (Fig. 1B). Supplementation of TSF331 and TSR332 significantly (^∗∗^*p* = 0.00811 and ^∗∗^*p* = 0.00223) reduced the UA levels to 3.57 mg/dL and 1.92 mg/dL, respectively. Remarkably, TSR332 reduced the UA level to a greater extent than that by TSF331, whereas La322 reduced UA to a level (4.62 mg/dL) comparable to that in the HP group (Fig. 1B, Table S1).

### *L. reuteri* TSR332 displays preventive and therapeutic effects in stabilizing the serum UA level in hyperuricemia model rats

To investigate the preventive and therapeutic effects of TSR332 in rats further, more treatment schemes were evaluated and longer treatment periods were used (Fig. 2). The outline of this animal study was presented as Fig. 2A. The blank control group was fed a regular diet, and 4 other groups were fed a high-purine diet to increase purine uptake from food. The symptom was induced by oxonate injection on day 7-14. One hyperuricemia group served as the symptom control (HP), and three other hyperuricemia groups were supplemented with probiotic strains for the designated periods (pretreatment on days 1–7, treatment on days 7–14, or probiotics on days 1–14). Blood serum was collected and tested for UA on days 0, 7, and 14. On day 0, serum UA levels were similar in all groups. After the beginning of potassium oxonate injection on day 7–14, UA levels on days 7 and 14 in the symptom control (HP) group (3.26 mg/dL; ^##^*p* = 0.00772 and 3.64 mg/dL; ^##^*p* = 0.00307, respectively) increased significantly as compared to the levels in the blank control group (1.77 mg/dL and 1.60 mg/dL, respectively) (Fig. 2B). UA levels were significantly lower in the pretreatment group than in the HP group on both day 7 and 14, and a more dramatic reduction was seen on day 7 (1.95 mg/dL, ^∗∗∗^*p* = 0.00088) than on day 14 (2.43 mg/dL, ^∗∗^*p* = 0.00921). UA levels were slightly lower in the treatment group (2.74 mg/dL) than in the HP group (3.26 mg/dL) on day 7 and were significantly lower on day 14 (2.26 mg/dL vs. 3.64 mg/dL, ^∗∗^*p* = 0.00815). On day 7, in the probiotics group, the UA levels (2.40 mg/dL) were significantly (^∗^*p* = 0.03084) lower than those in the HP group (3.26 mg/dL), and the difference increased further (2.38 mg/dL vs. 3.64 mg/dL, ^∗∗^*p* = 0.00721) on day 14 (Fig. 2B, Table S2).

### *L. reuteri* TSR332 stabilizes the UA level in acute hyperuricemia rat model

To investigate the therapeutic effects of TSR332 on acute hyperuricemic rats, hyperuricemia was induced using a high-purine diet and potassium oxonate injection for 14 days straight. Treatments were similar to those used for the preliminary test, except for the duration (Fig. 3A). The blank control group was fed a regular diet, and two other groups were fed a high-purine diet to increase purine uptake from food. One hyperuricemia group served as the symptom control (HP) and another hyperuricemia group was supplemented with probiotic TSR332 daily for 14 days. On day 0, UA levels were similar in all groups (Fig. 3B). The high-purine diet and potassium oxonate injection consistently and significantly induced high UA levels in the HP group on days 7 and 14 (3.73 mg/dL; ^##^*p* = 0.00705 and 3.60 mg/dL; *p* = 0.00519, respectively). When compared with the HP group, supplementation of TSR332 significantly decreased UA levels on days 7 and 14 (2.38 mg/dL; ^∗∗∗^*p* = 0.00091 and 2.35 mg/dL; ^∗∗^*p* <0.00127, respectively; Fig. 3B, Table S3).

Body weights increased over time in the control group (96.63 g to 214.37 g) and were significantly lower in the HP group (125.20 g; ^#^*p* = 0.0356 and 160.07 g; ^##^*p* = 0.00664, respectively) than in the control group on days 7 and 14, whereas no significant difference was detected between HP group and the TSR332 group (131.38 g and 170.13 g, respectively; Fig. 3C, Table S3). To evaluate the effect of TSR332 on kidney function, blood creatinine levels were analyzed and were found to vary between 0.18–0.35 mg/dL. Although some differences were seen among the groups, they were not significant (Fig. 3D, Table S3).

## Discussion

Recent studies have shown that some lactic acid bacteria strains can degrade purine nucleosides to purine bases, which are not as easily absorbed by intestinal cells as nucleosides (Yamada et al., 2016; Salati et al., 1984; Stow & Bronk, 1993). However, the purine degradation efficiency is strain-specific, not species-specific (Kano et al., 2018). In order to screen efficiently for purine-degrading strains, the in vitro purine assimilation assay is an essential primary step. Our results showed that the assimilation rates of different purines generally correlated in most of the evaluated strains. For instance, TSR332 and TSF331 had high inosine and guanosine assimilation rates. Interestingly, in a few strains, purine assimilation was selective. For instance, Bv-889 had a moderate inosine assimilation rate (25.63%) but exhibited a very high guanosine assimilation rate (78.98%). Most organisms exploit two strategies to obtain purine and pyrimidine nucleotides; they either synthesize them *de novo* through biosynthetic pathways, or they utilize preformed precursors present in the environment. In order to utilize exogenous nucleotide precursors, the cells need to transport these compounds across the cytoplasmic membrane. In studies of the uptake of nucleotide precursors, microorganisms have been found to encode several transport systems with different and overlapping affinities (Martinussen et al., 2010). Further research is needed to elucidate the selection mechanism behind purine metabolism in microorganisms.

The development of an animal model of hyperuricemia is challenging because commonly used laboratory animals, such as rats, mice, and rabbits, all express urate oxidase (Stavric & Nera, 1978; Lu et al., 2018). Potassium oxonate blocks the activity of hepatic uricase and, when combined with a high-purine diet, induces hyperuricemia in rats (Sanchez-Lozada et al., 2008). In our study, two strains, TSR332 and TSF331, stood out from the purine assimilation screening and showed the most robust capability to degrade purine without detectable UA production. This finding suggests the existence of probiotic strains that have active purine-metabolic systems. Furthermore, inosine and guanosine used in the assay were highly effectively converted into hypoxanthine and guanine, which indicates the presence of nucleosidase activity, converting nucleosides to purine bases. Nucleosidases are widely found in plants and microorganisms but have not yet been detected in mammals (Ogawa et al., 2006). Therefore, supplementation of the probiotic strains TSR332 and TSF331 may eliminate excess purine assimilation in the digestive tract through their nucleosidase activity.

Even though only 3 out of 24 strains screened in the in vitro degradation assay were selected for further study in rats, the in vitro and in vivo tests produced corresponding results. In the 8-day preliminary test in animals, TSR332 and TSF331 displayed the best UA-lowering effect, whereas La322 had no noticeable effect. Therefore, the purine assimilation assay is an efficient and reliable screening method for highly efficient purine-degrading strains (Shen, Shang & Li, 2011; Sugimura, Hagi & Hoshino, 2011; Elmadfa, Klein & Meyer, 2010; Turpin et al., 2010). Although only the most effective strain, TSR332, was selected for further study of its preventive and therapeutic effects, the other two strains, Bv-889 and TSF331, are worth further investigation.

Unlike current drugs, TSR332 showed a hyperuricemia-preventive effect, which is the most attractive finding of this study (Luo et al., 2020; Zhang et al., 2020). According to the experimental scheme, rats in the pretreatment group were healthy and free from hyperuricemia during the period of probiotic administration. The symptom was induced by the end of probiotic intervention, and the supplementary of TSR332 was discontinued after the symptom induction. Even though probiotic treatment was not present at the same time as hyperuricemia induction, the UA-lowering effect was surprisingly the strongest at the beginning of hyperuricemia induction on day 7. Although the effect decreased over time and showed a similar effect with other two groups on day 14, our results indicate that TSR332 has a protective effect that may offer new options for UA management. In the treatment group, the symptom was induced at the same time as the probiotic intervention. Even though the UA-lowering effect was mild on day 7, the effect increased along with the supplementary of TSR332 over time on day 14. The beneficial effect of TSR332 seems to be influenced by hyperuricemia induction at first; however, its efficacy gradually catches up with time. In acute hyperuricemic rats, the long-term TSR332 treatment showed a stable UA-lowering effect, with no noticeable side effects in terms of body weight and kidney function.

## Conclusions

Three probiotic strains, *L. reuteri, L. fermentum*, and *L. plantarum,* used in this study are common probiotic microorganisms that are used in yogurt, fermented milk products and food supplements. Besides, the infection potential of the genus, *Lactobacillus*, is mainly non-pathogenic or some opportunistic infection, which usually occurs in the immunocompromised patient (Peivasteh-Roudsari et al., 2019). In our study, the supplementary of the probiotic *Lactobacillus* strain TSR332 was effective for UA control in rats and showed no negative effects on body weight and creatinine levels. Taken all together, the probiotic treatment may be safe to use in hyperuricemia patients, and thus may have clinical potential. Although the result can be different in animal and human studies, further studies are still warranted to evaluate whether TSR332 exerts similarly beneficial effects in humans and to investigate the underlying mechanism (Zhou et al., 2020).

##  Supplemental Information

10.7717/peerj.11209/supp-1 Supplemental Information 1 Raw data of Figs. 1, 2, 3 and 5 (1) Preliminary tests of TSF331, TSR332, and La322 in hyperuricemic rats. (2) Pretreatment and treatment effects of TSR332 in hyperuricemic rats. (3) UA, body weight, and kidney function in severe hyperuricemic rats. (5) In vitro purine assimilation assays of inosine and guanosine in probiotic strains.

## Additional Information and Declarations

### Data Availability

The following information was supplied regarding data availability: The raw data for Figs. 1– 3 and 5 are available in the Supplementary Tables.
